# Anodal transcutaneous spinal direct current stimulation influences the amplitude of pain-related evoked potentials in healthy subjects

**DOI:** 10.1038/s41598-023-47408-x

**Published:** 2023-11-27

**Authors:** Frederic Eberhardt, Elena Enax-Krumova, Martin Tegenthoff, Oliver Höffken, Özüm Simal Özgül

**Affiliations:** Department of Neurology, Berufsgenossenschaftliches Universitätsklinikum Bergmannsheil GmbH, Ruhr-University Bochum, Bürkle-de-la-Camp-Platz 1, 44789 Bochum, Germany

**Keywords:** Neural circuits, Sensory processing, Somatosensory system, Neurology

## Abstract

It has already been described that transcutaneous spinal direct current stimulation (tsDCS) can selectively influence nociceptive evoked potentials. This study is the first aiming to prove an influence of tsDCS on pain-related evoked potentials (PREP) using concentric surface electrodes (CE), whose nociceptive specificity is still under discussion. 28 healthy subjects participated in this sham-controlled, double-blind cross-over study. All subjects underwent one session of anodal and one session of sham low-thoracic tsDCS. Before and after the intervention, PREP using CE, PREP-induced pain perception and somatosensory evoked potentials (SEP) were assessed on the right upper and lower limb. We found a decrease in PREP amplitude at the lower limb after sham stimulation, but not after anodal tsDCS, while SEP remained unchanged under all studied conditions. There was no difference between the effects of anodal tsDCS and sham stimulation on the studied parameters assessed at the upper limb. PREP-induced pain of the upper and lower limb increased after anodal tsDCS. The ability of influencing PREP using a CE at the spinal level in contrast to SEP suggests that PREP using CE follows the spinothalamic pathway and supports the assumption that it is specifically nociceptive. However, while mainly inhibitory effects on nociceptive stimuli have already been described, our results rather suggest that anodal tsDCS has a sensitizing effect. This may indicate that the mechanisms underlying the elicitation of PREP with CE are not the same as for the other nociceptive evoked potentials. The effects on the processing of different types of painful stimuli should be directly compared in future studies.

## Introduction

Spinal cord stimulation (SCS) using electrical currents applied through epidural electrodes is an established method for neuromodulation in pain treatment^1^. Transcutaneous spinal direct current stimulation (tsDCS) has been investigated in the last years to explore whether clinically relevant effects can also be achieved in a non-invasive way, making it a potential therapeutic intervention. In tsDCS, neuromodulation is achieved by a direct current flowing between two electrodes placed on the patient's skin. One of these electrodes is placed over the spinal cord, while a second electrode is placed in a location distant from the spine^2^.

Previous studies show heterogenous results regarding the effects of tsDCS on nociception. Some studies did not find a significant effect on pain perception^3–5^, one study has reported effects on pinprick pain, but not on pain caused by single electrical pulses^6^, one other described an effect on the perception of the last stimulus of a series of five electrical stimuli applied on the skin at 2 Hz, but not on the perception of the first stimulus of the series^7^. However, several studies have reported effects on different painful stimuli and pain-related values such as pinprick stimuli^6,8^, painful cutaneous electrical stimulation^7,9^, cold pressor test^2^, pressure pain^10^, and on the nociceptive flexion reflex^9^. Two studies have also examined the effects of anodal tsDCS on pain intensity in patients with neuropathic pain^3,11^. By using laser-evoked potentials (LEP) it was possible to investigate the influence of tsDCS on cerebral potentials reflecting the specific excitation of nociceptive fibers^2,4,8^. Truini et al.^2^ reported that anodal tsDCS applied to the spinal cord at low-thoracic level reduces the magnitude of the cortical recorded N2 wave of LEP after stimulation of the foot dorsum, while responses after stimulation of the face remained unchanged^2^. Lenoir et al.^4^ reached similar results for anodal tsDCS at low-thoracic level, showing a decrease of the magnitude of the N2 wave of LEP after foot stimulation, but not after hand stimulation and no effect of anodal tsDCS at cervical level. The cerebral responses after non-nociceptive stimulation using short-lasting mechanical vibration to selectively activate Aß-fibers were not affected. Based on that research it has been hypothesized that anodal tsDCS induces changes at the level of the stimulated spinal segments near the anode, which results in decreased amplitudes of LEP^2,4^. For anatomical reasons, this appears reasonable since the site of tsDCS stimulation is near the spinal segments which contain the synaptic relays between the first-order neurons and the second-order neurons of the spinothalamic pathway, transmitting nociceptive signals from the lower limb to the brain. In contrast, the neurons involved in transmitting nociceptive signals from the upper limb or from the face to the cortex are located at much greater distances from the tsDCS stimulation site. Moreover, the dorsal column-medial lemniscus pathway has no synaptic relays in the spinal cord, so that by this reasoning, SEP should not be influenced by low thoracic tsDCS.

This specific effect for nociception raised the question if pain-related evoked potentials (PREP) using concentric surfaces electrodes (CE) could be similarly influenced by tsDCS, although their specificity for the spinothalamic pathway is still being discussed^12–14^. It has been reported that PREP using CE allow the evaluation of electrophysiological stimulus transmission after stimulating intraepidermal small thin- and unmyelinated fibers, mainly the Aδ-fibers^15–19^. A specially designed concentric electrode (K2-electrode)^16^ with a small anode–cathode distance resulting in a high current density despite low current intensities is being used for PREP recordings. Thus, only the nerve fiber endings of nociceptive fibers within the superficial skin layers are being depolarized, whilst avoiding activation of Aβ-fibers in deeper layers.

Following the study of Lenoir et al., we designed a double-blind controlled randomized cross-over study with two sessions per subject: one session of low thoracic anodal tsDCS compared to one session of sham tsDCS to examine the effects of anodal tsDCS on PREP using CE. Additionally, we analyzed the effect on somatosensory evoked potentials (SEP). Our hypothesis was that the low thoracic anodal tsDCS can reduce both the subjective pain intensity and the amplitudes of the PREP with CE of the lower limb, but not the upper limb and has no effect on SEP^2,4^.

## Methods

The study was approved by the local ethic committee of the Faculty of Medicine, Ruhr-Universität Bochum (Reg. Nr. 20-6929, 27.4.2020) and carried out according to the Helsinki Declaration. Written informed consent was obtained from all participants.

### Participants

During the time from July 2020 to January 2021, we recruited 28 healthy volunteers, older than 18 years. Subjects with relevant medical conditions (e.g., diabetes, migraine, pacemaker, psychiatric or neurological diseases), recent use of local anesthetics, topical capsaicin, anticonvulsants or antidepressants, as well as subjects with substance abuse or current pain were excluded.

### Procedure

The subjects were randomly assigned to eight groups according to the order of testing by limb (foot first/hand first), by test method (PREP first/SEP first) and by order of stimulation types (sham stimulation first/anodal tsDCS first). Subjects and examiner were blinded to the order of stimulation types, but not to order of testing by limb and test method.

**Figure 1:**
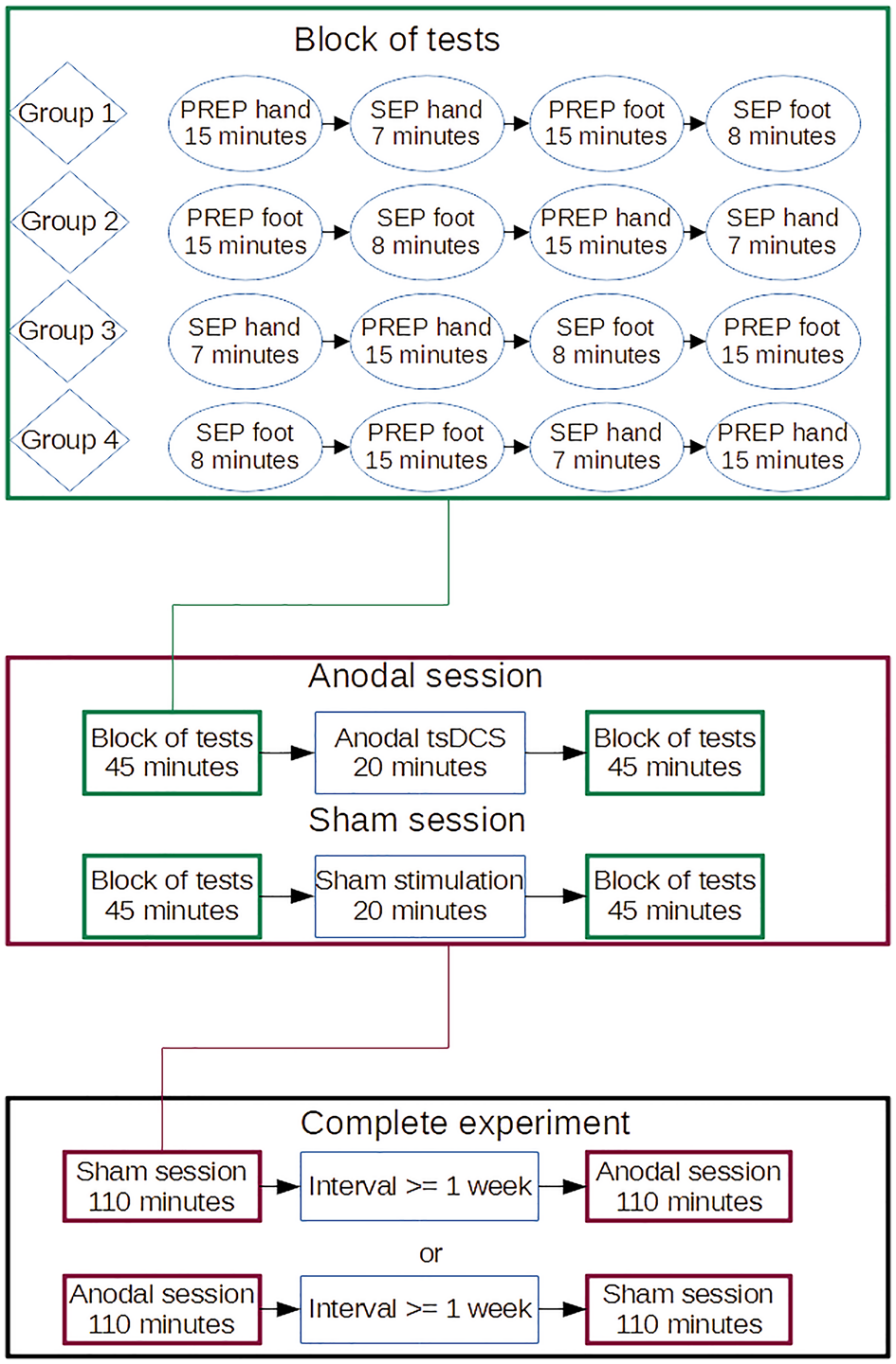
Experiment design. Each subject participated in a sham session and an anodal session, which each consisted of one block of tests before and after sham stimulation or anodal tsDCS. The order of measurements in a block of tests depended on the group the subject was in. The top part of the figure shows the timeline for one block of tests. The middle part shows the timeline for one session, which consisted of either sham or anodal tsDCS, preceded and followed by one block of tests. The bottom part shows the timeline for the whole experiment, which consisted either of one session of anodal tsDCS, followed by an interval of at least one week and then one session of sham tsDCS, or of one session of sham tsDCS, followed by an interval of at least one week and then one session of anodal tsDCS.

The experiment was divided in two sessions, which were at least one week apart. On each subject, anodal tsDCS was performed in one session and sham stimulation in the other session. Before and after application of tsDCS in each session, SEP and PREP using CE were performed on right hand and right foot of each subject. The sequence of recording PREP and SEP after electrical hand and foot stimulation was randomly assigned to 4 different groups (block of tests). The different measurements in each session were performed immediately after each other and also immediately after the end of tsDCS, only with a delay of a few minutes necessary to prepare the next measurement. Setup and timeline of the experiment are visualized in Fig. 1. At the start of each session, the EEG electrodes required for all measurements were attached at Cz, Fz, CP3, and at the earlobes, according to the international 10–20 system.

During the experiment, the subjects were seated in an armchair. They were instructed to keep their gaze fixed on a mark at a distance of about 1.5 m in front of them during all measurements, in order to reduce artifacts due to eye movements, similar to previous studies, e.g. ^4^.

#### Somatosensory evoked potentials (SEP)

SEP was recorded by stimulation of the right median nerve (hand) and the right tibial nerve (foot), using a block electrode, which was fixed to the stimulation site with a velcro strap. The used parameters were in accordance with standard protocols recommended in the literature^20^.The distance between anode and cathode of the block electrode was 20 mm. For median nerve SEP, the cathode was placed between the tendons of the palmaris longus and flexor carpi radialis muscles, approximately 2 cm proximal to the wrist crease, the anode distal. For tibial nerve SEP, the cathode was placed midway between the medial border of the Achilles tendon and the posterior border of the medial malleolus, while the anode was positioned 20 mm more distal. This followed standard protocols recommended in the literature^20^, except that due to the block electrodes used in our experiment, the distance between anode and cathode was always 20 mm in our study. The correct positioning of the block electrode over the carpal tunnel or behind the medial malleolus was checked before every SEP measurement block. Stimulation was performed with a frequency of 3 Hz (median nerve) or 2 Hz (tibial nerve). Before every SEP measurement, the current intensity was set to reliably induce a visible muscle contraction. Each stimulus lasted 0.1 ms. Each SEP consisted of 200 stimuli. To record the SEP, we used CP3 for the median nerve SEP and Cz for the tibial nerve SEP as the recording electrodes, while Fz was used as reference.

#### Pain-related evoked potentials (PREP) using concentric surface electrodes

The procedure followed previous protocols^12,13,21^ with slight modifications. PREP was performed on the area innervated by the right radial nerve (hand dorsum) and the right sural nerve (foot, behind the malleolus lateralis). For PREP in the area of the radial nerve, the concentric electrode was placed between the basis of the metacarpal bones of thumb and index finger. For PREP in the area of the sural nerve, the concentric electrode was placed midway between the lateral border of the Achilles’ tendon and the posterior border of the lateral malleolus. For the stimulation, we used a planar concentric electrode with the same specifications as previously described^16^. The electrode was fixed to the stimulation area using an elastic band-aid tape. We adjusted detection threshold (DT) and pain threshold (PT) by increasing current intensity starting with 0.2 mA steps until subjects reported a tingling sensation (DT) or a pinprick-like pain (PT). Then we proceeded in 0.1 mA steps in a randomized order above or below the supposed thresholds until subjects reported a stable perception. To elicit evoked potentials, we applied 40 stimuli with an interstimulus interval of 13–17 s and current intensity at the twofold of the individual pain threshold. Each stimulus consisted of 3 successive monopolar square waves, each with a duration 200 µs and an inter-wave interval 5 ms. After every five stimuli, subjects were asked to rate the perceived pain of the last five stimuli on a numerical rating scale (NRS) from 0 to 100, with 0 being no pain and 100 being the strongest imaginable pain, to document the elicited pain during electrical stimulation for PREP-recording. To record the PREP Cz was used as recording electrode, while electrodes attached to the earlobes were used as reference electrode.

#### Transcutaneous spinal direct current stimulation (tsDCS)

For tsDCS, two saline-soaked sponge electrodes (7 × 5 cm) were fixed to the volunteers' skin. The cathode was fixed to the right shoulder, while the anode was fixed to the spinous process of thoracic vertebra 10 (Th10). The stimulation device was a DC-stimulator produced by neuroConn GmbH, Ilmenau, Germany. For anodal tsDCS, a direct current stimulation increased gradually over a timespan of 10 s to an intensity of 2.5 mA, at which it was kept up for 20 min and then was gradually reduced to zero over a timespan of 20 s. For a sham stimulation, the current also increased gradually over a timespan of 10 s, but was immediately after reduced to zero during a timespan of 20 s. In both conditions, the electrodes were removed after the device signaled that 20 min of stimulation were over. Both stimulations do not differ in evoked sensation, so that neither the subjects nor the examiner could differentiate sham stimulation from anodal tsDCS. Before starting tsDCS, the device checked the impedance between the electrodes and started not before the impedance was reliable below 50 kOhm. The tsDCS parameters were identical to those used by Lenoir et al.^4^, with the only difference that we did not additionally use a cervical electrode.

### Data analysis

Storage for offline analysis was achieved by a 32-channel-amplifier (Brain Amp, Brain Products, Germany; Bandwidth: 0.1 Hz–1 kHz; digitization sampling rate: 5 kHz). Employing Vision Recorder Version 1.03 offline analysis was carried out. For this, the data was segmented into epochs from 200 ms before and 800 ms after stimulus onset for data acquired during PREP measurements and epochs ranging from 30 ms before stimulus onset to 80 ms after stimulus onset for data acquired during SEP measurements.

Following previous studies for PREP using CE^19,22^, a slightly wider bandpass filter of 0.5–1000 Hz and a notch filter at 50 Hz were used. To reduce a bias caused by a startling response, the first epoch of every PREP measurement was removed from further analysis^16,17,22^. Before averaging, for each epoch a baseline correction using the pre-stimulus interval of 200 ms was applied.

40 stimuli were applied intending to make the potential of PREP as clear to detect as possible. After data from ongoing studies in the neurological department of the Bergmannsheil Bochum seemed to indicate a decrease of the N1P1-amplitude when using 40 stimuli (data not shown), we split the data into the epochs 2–20 (first half) and 21–40 (second half). The comparison showed that the data of the second half contained more artifacts due to body movements and blinking, as well as significantly smaller amplitudes (data not shown). Therefore, the second half of the data was discarded and only the average of epochs 2–20 was used in later analysis, as was previously the practice^22,23^.

In the PREP measurement we determined the N1- and P1-peaks, calculated the N1P1-amplitude and documented the N1-latency of each potential, as described in previous studies^16,19^. Following previous studies^18,19^, we defined the N1-peak as the first negative peak after application of the stimulus followed by the most positive peak, defined as the P1-peak.

In the SEP measurements, the evaluated potentials depended on the examined limb. For the upper limb (median nerve SEP), the amplitude of the N20-potential was subtracted from the amplitude of the P25-potential, resulting in the N20-P25-amplitude, and the latency of the N20-potential was measured. For the lower limb (tibial nerve SEP), the same operations were performed with the N30- and P40-potentials.

Subjects with EEG recordings in which the N1-, P1- N20-, P25, N30- or P40-potentials could not be clearly identified due to artifacts (e.g., movement or electrode artifacts) during at least one of the time points of the study, were excluded from further analysis.

#### Statistical analysis

Before testing for effects of tsDCS modality, Shapiro–Wilk-tests for normal distribution, as well as tests for group effects and carry-over effects were performed. Because not all variables were normally distributed in all measurements, nonparametric tests were used to check for group effects, comparing 4 groups denoted in Fig. 1 as group 1–4 (hand first/foot first × PREP first/SEP first). For this, a total of 8 Kruskal–Wallis-tests were performed (anodal/sham tsDCS × before/after tsDCS × foot/hand), resulting in a Bonferroni-corrected level of significance of 0.00625. Each of these tests compared the dependent variables between the 4 groups.

After this, tests for carry-over-effects were performed. For this, we followed a procedure recommended in the literature^24^: For each subject, the data from the first measurement of each kind (PREP lower limb, PREP upper limb, median nerve SEP, tibial nerve SEP) were summed up for the sham stimulation session and the anodal tsDCS session (e.g. N1P1-amplitude of PREP hand in the sham session, before sham tsDCS + N1P1- amplitude of PREP hand in the anodal session, before anodal tsDCS). The subjects were then split into two groups, depending on whether they had received anodal tsDCS or sham tsDCS in their first session. For each of these groups, the average value of the sums described above was compared for each dependent variable. For this, two Kruskal–Wallis-tests were performed, one for the data from the upper limb and one for the data from the lower limb, resulting in a Bonferroni-corrected level of significance of 0.025.

To evaluate effects of tsDCS modality on the different dependent variables, the values measured before tsDCS were first subtracted from the values measured after tsDCS in order to obtain the difference. The difference for each measured quantity (N1P1-amplitude, N1-latency, NRS value of the PREP-induced pain, DT, PT for PREP and NP-amplitude, N-latency, current intensity used for SEP) was then used as dependent variable in an ANOVA with the independent variables 'stimulation' (sham stimulation or anodal tsDCS) and 'limb' (lower or upper limb) in order to evaluate how the dependent variables changed during an experimental session depending on stimulation type, for upper and lower limb. The decision to use an ANOVA although not all variables were normally distributed for all measurements was made because the number of subjects was seen as large enough to guarantee a sufficient robustness to deviations from normal distributions, which is supported by previous studies^25^.

## Results

The demographic data of the examined subjects are presented in Table 1. 3 subjects were excluded because of artifacts in the EEG recording. The remaining 25 subjects were included in the data analysis. For a comparison of the demographics before and after subject exclusion see Table 1.

**Table 1:** Subjects demographics.

	before exclusion	after exclusion
# of subjects	28	25
# of female subjects	16	15
age [years] ± SD; min–max	24.8 ± 4.2; 18–35	24.2 ± 3.8; 22–34
height [cm] ± SD; min–max	172.5 ± 8.3; 159–193	171.3 ± 8.0; 159–180

We tested our data for normal distribution, group effects depending on the randomization order, and carry-over effects, as described in the methods section. For a detailed description of the results of the tests for group effects see Table 2, for the results of the tests for carry-over-effects see Table 3. No group effects or carry-over effects were found, but not all of the data was normally distributed. In Table 4, we present the mean value and standard deviation for the assessed variables in all conditions (lower limb/upper limb, sham stimulation/anodal tsDCS, before stimulation/after stimulation).

**Table 2:** Results of the Kruskal–Wallis tests for group effects depending on randomization order.

			N1P1 amplitude		N1 latency		NRS value		Detection threshold		Pain threshold		N latency (SEP)		NP amplitude (SEP)		Current intensity used for SEP	
			H	p	H	p	H	p	H	p	H	p	H	p	H	p	H	p
Sham	Pre	Foot	2.845	0.416	2.52	0.472	1.56	0.668	6.014	0.111	6.122	0.106	2.371	0.499	6.178	0.103	4.513	0.211
		Hand	4.374	0.224	4.309	0.23	5.395	0.145	4.798	0.187	7.640	0.054	5.493	0.139	1.314	0.726	2.782	0.426
	Post	Foot	3.627	0.305	2.642	0.45	1.585	0.663	4.203	0.24	5.119	0.163	2.324	0.508	2.309	0.511	2.806	0.423
		Hand	6.418	0.093	3.591	0.309	6.88	0.076	5.281	0.152	6.759	0.08	6.82	0.078	0.647	0.885	2.098	0.552
Anodal	Pre	Foot	5.79	0.122	2.36	0.501	4.472	0.215	0.52	0.915	2.156	0.541	5.254	0.154	1.129	0.77	3.437	0.329
		Hand	8.704	0.034	5.78	0.123	2.692	0.442	3.334	0.343	4.694	0.196	1.307	0.727	0.143	0.986	5.624	0.131
	Post	Foot	6.196	0.102	2.785	0.426	4.71	0.194	1.802	0.614	1.9	0.593	3.612	0.306	4.091	0.252	3.314	0.346
		Hand	1.836	0.607	1.874	0.599	2.681	0.444	2.51	0.474	5.777	0.123	3.142	0.37	0.571	0.903	4.233	0.237

**Table 3:** Results of the Kruskal–Wallis tests for carry-over effect.

	N1P1 amplitude		N1 latency		NRS value		Detection threshold		Pain threshold		N latency (SEP)		NP amplitude (SEP)		Current intensity used for SEP	
	H	p	H	p	H	p	H	p	H	p	H	p	H	p	H	p
Foot	1.305	0.253	3.322	0.068	0.24	0.624	1.858	0.173	0.394	0.53	0.107	0.743	0.426	0.514	0.074	0.786
Hand	0.145	0.703	0.19	0.663	0.54	0.463	0.074	0.785	0.297	0.586	3.054	0.081	0.358	0.55	2.673	0.102

**Table 4:** Dependent variables before and after tsDCS.

	Foot				Hand			
	Sham		Anodal		Sham		Anodal	
	Before stimulation	After stimulation	Before stimulation	After stimulation	Before stimulation	After stimulation	Before stimulation	After stimulation
N1P1 amplitude[µV]	35.0 ± 14.4	26.5 ± 13.5	32.1 ± 14.6	31.7 ± 11.3	38.5 ± 19.5	34.2 ± 13.9	41.3 ± 15.9	34.3 ± 17.6
N1 latency [ms]	172.8 ± 30.4	174.7 ± 32.5	171.4 ± 35.8	168.9 ± 39.6	141.4 ± 18.9	144.7 ± 19.9	143.4 ± 18.7	146.2 ± 26.0
PREP-induced pain intensity [NRS (0–100)]	35.5 ± 26.1	32.8 ± 25.2	32.1 ± 24.7	36.1 ± 25.8	24.9 ± 20.9	22.6 ± 20.3	26.7 ± 22.2	27.5 ± 22.6
DT [mA]	0.87 ± 0.26	0.93 ± 0.41	0.86 ± 0.49	0.85 ± 0.26	0.66 ± 0.33	0.66 ± 0.27	0.64 ± 0.2	0.69 ± 0.26
PT [mA]	1.02 ± 0.55	1.01 ± 0.47	0.95 ± 0.49	0.94 ± 0.32	0.79 ± 0.42	0.71 ± 0.3	0.73 ± 0.28	0.76 ± 0.31
NP amplitude SEP [µV]	1.95 ± 1.15	2.06 ± 1.21	1.92 ± 1.01	1.93 ± 1.08	3.82 ± 1.54	3.87 ± 1.88	3.27 ± 1.74	3.41 ± 1.67
N latency SEP [ms]	30.4 ± 3.0	30.56 ± 3.12	31.4 ± 3.9	30.68 ± 3.59	18.0 ± 1.1	18.44 ± 0.96	18.1 ± 1.3	18.36 ± 1.38
Current intensity used for SEP [mA]	17.8 ± 9.8	21.0 ± 10.4	17.4 ± 7.5	19.1 ± 9.1	6.4 ± 3.3	6.7 ± 3.5	6.5 ± 2.1	6.3 ± 2.0

**Figure 2:**
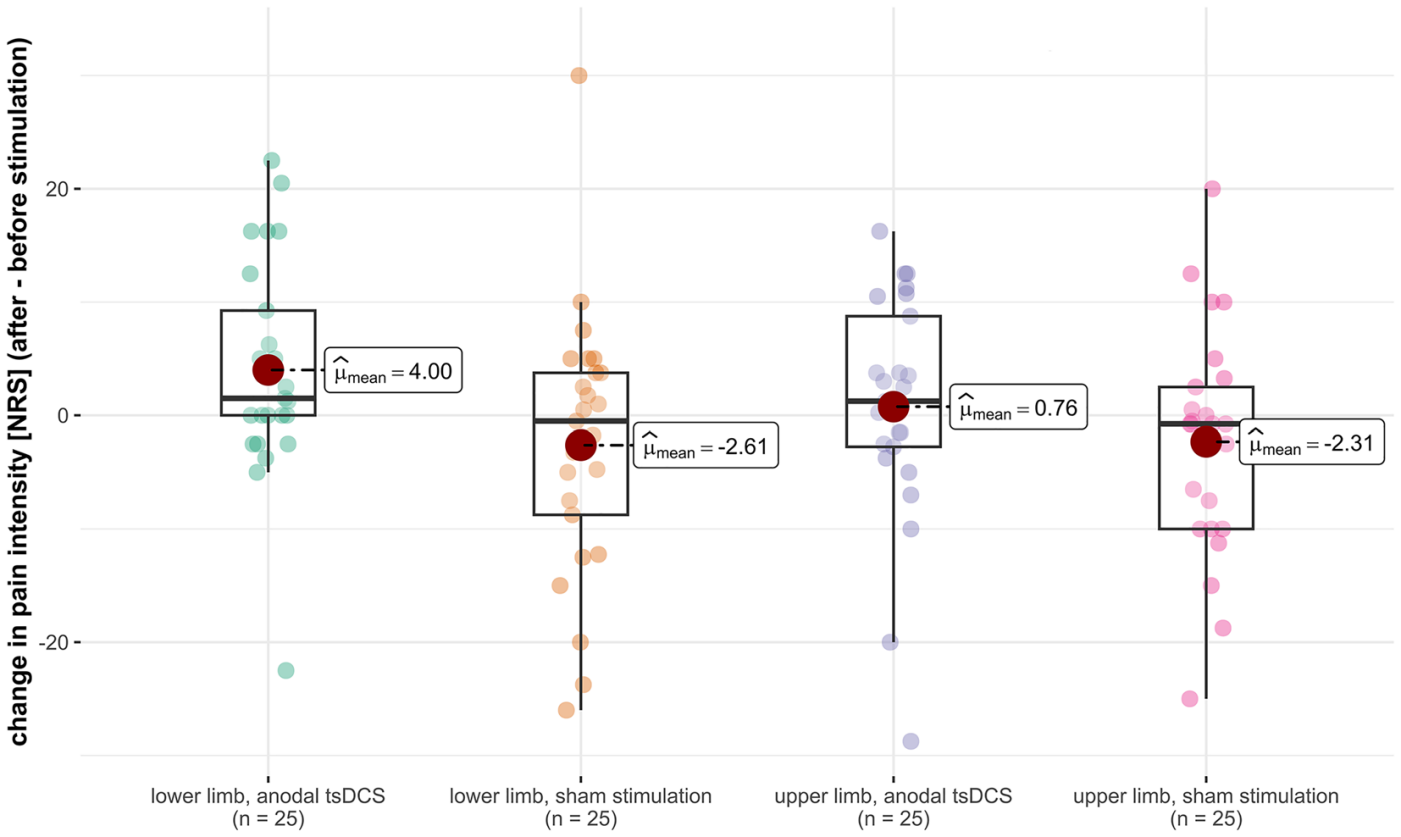
Effect of tsDCS on pain intensity measured by NRS. The red circles denotes the mean, the horizontal line through each box denotes the median. The whiskers denote 1.5× the interquartile distances.

**Figure 3:**
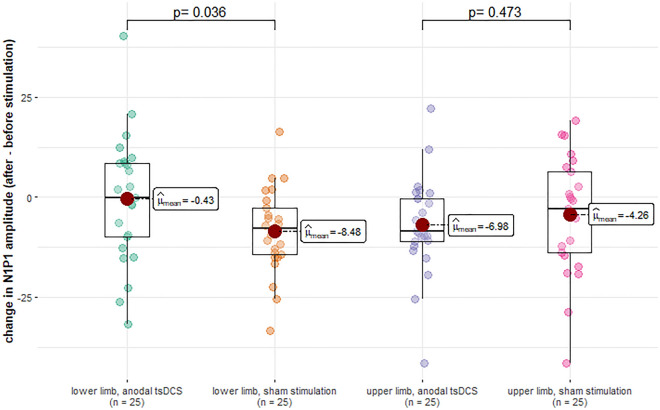
Effect of tsDCS on N1P1 amplitude. The red circles denote the mean, the horizontal line through each box denotes the median. The whiskers denote 1.5× the interquartile distances.

An ANOVA with the independent variables ‘stimulation’ (sham stimulation or anodal tsDCS) and ‘limb’ (lower or upper limb) and the dependent variables ‘N1P1-amplitude’, ‘N1-latency’, ‘pain intensity’, ‘detection threshold’, ‘pain threshold’, ‘NP-amplitude of SEP’, ‘N-latency of SEP’ and ‘current intensity used for SEP’ found the following effects (for a list of all results see Table 5):

1. An effect of ‘limb’ (lower vs. upper limb) on ‘current intensity used for SEP’ (*p* = 0.007, η^2^ = 0.073). This shows that the intensity of the current flowing through the SEP stimulation electrode was different between the upper and the lower limb. Since Shapiro–Wilk-tests showed that the values were not normally distributed (*p* < 0.001 for upper as well as lower limb), we used a Mann–Whitney-U-test with the groups ‘lower limb’ and ‘upper limb’ for post-hoc-testing. This test found a significant difference (Mann–Whitney U = 597.0, Z = − 10.763, *p* < 0.001). Considering the median current intensities (17 mA for tibial nerve SEP, 6 mA for median nerve SEP), this shows that throughout the study, a significantly higher current intensity was used for the stimuli of the tibial nerve SEP than for the stimuli of the median nerve SEP.
2. An effect of ‘stimulation’ (tsDCS vs. sham) on ‘pain intensity’ (*p* = 0.023, η^2^ = 0.052). Analyzing the effects on the lower and upper limbs together, the pain intensity based on the numerical rating scale (NRS; 0–100) decreased on average by 2.46 after sham stimulation, while in a session with anodal tsDCS, it increased by 2.38. A Mann–Whitney-U-Test confirmed that this difference was significant with a medium effect size (*p* = 0.014, η^2^ = 0.061). On the other hand, no effect of the interaction 'limb' x 'stimulation' on the NRS value was found. In Fig. 2, we give a visualization of this result. As demonstrated, while only a main effect of the type of stimulation on the pain intensity was found, the effect on the pain intensity caused during PREP in the area of the lower limb still descriptively seems to be more pronounced than the effect on the pain intensity caused during PREP in the area of the upper limb. After anodal tsDCS, the average NRS rating increased by 4.0 points for PREP in the area of the lower limb, while it only increased by 0.8 points for PREP in the area of the upper limb. After sham stimulation, the average NRS rating decreased by 2.6 points for PREP in the area of the lower limb, and by 2.3 points for PREP in the area of the upper limb.
3. An effect of the interaction between 'limb' (lower vs. upper limb) and 'stimulation' (tsDCS vs. sham) on the N1P1-amplitude (*p* = 0.047, η^2^ = 0.041). After sham stimulation both the N1P1-amplitudes of PREP after stimulation on the upper limb and lower limb decreased (upper limb on average by 4.26 µV, lower limb on average by 8.48 µV). After anodal tsDCS, N1P1-amplitudes of PREP after stimulation on the upper limb decreased as well on average by 6.98 µV, N1P1-amplitudes of PREP after stimulation on the lower limb show a smaller change as a decrease on average by only 0.43 µV. A post-hoc simple effects analysis found a significant difference with a small effect size between sham stimulation and anodal tsDCS for the lower limb (*p* = 0.036, η^2^ = 0.047), but not for the upper limb (*p* = 0.473, η^2^ = 0.005). This result is represented in Fig. 3.

**Table 5:** Results of the ANOVA for effects of limb and stimulation type.

	N1P1 amplitude		N1 latency		NRS value		Detection threshold		Pain threshold		N latency (SEP)		NP amplitude (SEP)		Current intensity (SEP)	
	F	p	F	p	F	p	F	p	F	p	F	p	F	p	F	p
Limb	0.192	0.662	0.417	0.52	0.494	0.484	0.002	0.96	0.058	0.811	1.421	0.236	0.066	0.798	7.735	0.007
Stimulation	0.996	0.321	0.214	0.645	5.36	0.023	0.033	0.856	0.544	0.463	1.088	0.3	0.005	0.947	1.246	0.267
Limb × stimulation	4.068	0.047	0.143	0.706	0.717	0.399	1.02	0.315	0.589	0.445	0.555	0.458	0.34	0.561	0.434	0.512

In contrast, we did not find an effect of 'stimulation' on any of the SEP values or an effect of an interaction of 'stimulation modality' with any other independent variable on any of the SEP values.

**Figure 4:**
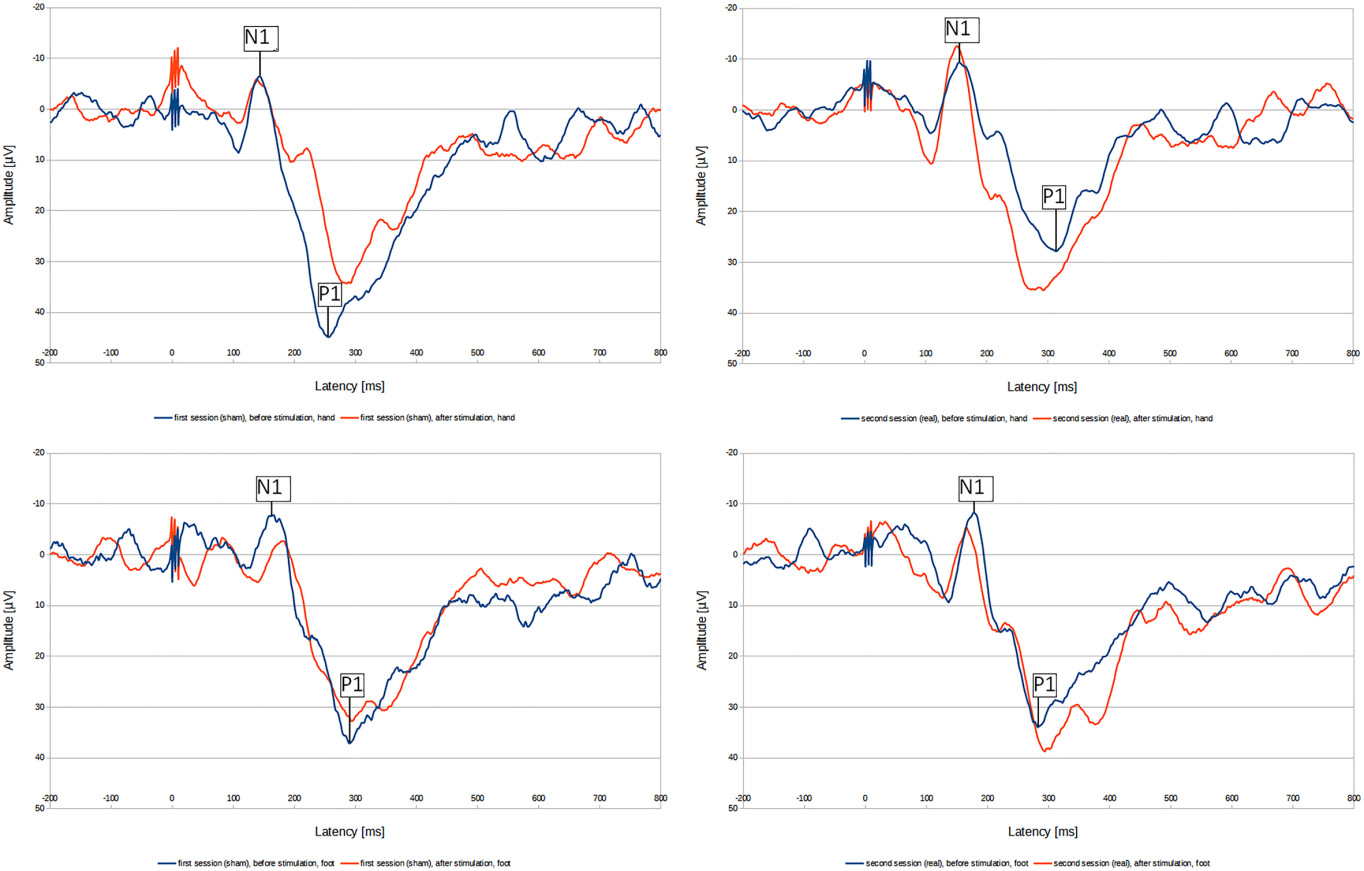
Typical PREP waveforms evoked by electrical stimulation of the upper and lower limb. The blue lines show the waveforms before tsDCS stimulation, the red lines after tsDCS stimulation. Top row: PREP evoked by CE stimulation of the upper limb. Bottom row: PREP evoked by CE stimulation of the lower limb.

In Fig. 4, we present the PREP of one subject from both sessions, for the upper and lower limb.

**Figure 5:**
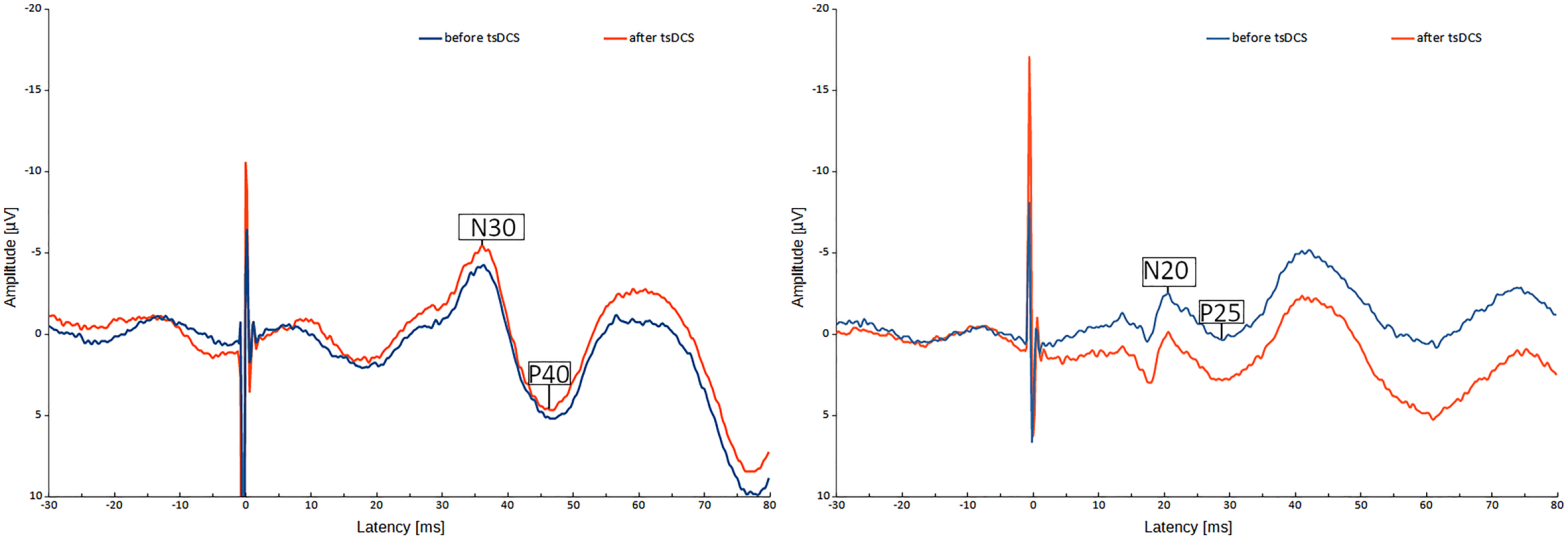
Typical SEP waveform. Left: tibial nerve SEP, right: median nerve SEP. The blue line shows the waveform before tsDCS stimulation, the red line after tsDCS stimulation.

In Fig. 5, we present a median nerve SEP and a tibial nerve SEP before and after tsDCS.

## Discussion

The present study found an effect of anodal low-thoracic tsDCS on PREP using CE, but it was not an inhibitory effect as excepted. While the N1P1-amplitude of PREP decreased under all other measuring conditions, it remained unchanged after low-thoracic anodal tsDCS when stimulating the lower limb. This missing decrease may be interpreted as a kind of suspended habituation effect or a sensitization, respectively. Since it has already been described that anodal tsDCS induces a local modulation of synaptic efficacy at the spinal cord level, the fact that there was an effect on PREP with CE but not on SEP would indicate that PREP is a nociceptive stimulus that is transmitted via the spinothalamic tract.

### Effect of limb on current intensity used for SEP

Our results showed a significant effect of the independent variable “limb” on the dependent variable “current intensity used for SEP”. Further analysis showed that the current intensity used in tibial nerve SEP was significantly higher than that used in median nerve SEP. Since the current intensity was set to reliably produce a muscle contraction, this result indicates that a higher current intensity was needed to fulfill this criterion over the tibial nerve than over the median nerve. This is most likely due to anatomical reasons, since the median nerve is located more superficially at the stimulation site than the tibial nerve.

### Effect of low-thoracic tsDCS on SEP

The study results show no effect of stimulation modality on any of the SEP values, which was in accordance to our hypothesis and underlines the validity of the significant findings regarding the effects on PREP parameters. This additional evidence that anodal tsDCS does not significantly affect the spinal transmission of non-nociceptive somatosensory stimuli is in line with the results of Lenoir et al.^4^ This study among other things compared the effect of low-thoracic anodal tsDCS, cervical anodal tsDCS and sham tsDCS on SEP caused by vibrotactile stimulation of the left and right third fingertips and the hallux. It found no effect on the amplitude and of latency on the N2- or P2-potentials. The explanation proposed by the authors was that since the dorsal column has its first synaptic relay in the medulla oblongata, effects on the synapses in the spinal cord segment under the anode do not directly affect the processing of somatosensory stimuli through the dorsal column. In contrast, Cogiamanian et al., 2008 found an effect on the cervicomedullary P30 component of the tibial nerve SEP, but no effect on cortical P39 potentials^26^. It should however be noted that these findings were based on an ANOVA with data of five participants.

### Effect of low-thoracic anodal tsDCS on N1P1-amplitudes of PREP using CE

In contrast to the SEP, we were able to demonstrate an effect of low-thoracic tsDCS on N1P1-amplitudes of PREP using CE after stimulation of the lower limb. This difference suggests that signal transmission after electrical stimulation with CE must be different from that via Aß-fibers stimulated by SEP electrodes. Lenoir et al.^4^ interpreted the effect of low-thoracic tsDCS on LEP after laser stimulation of the lower limb as a modulation of synaptic efficacy on spinal level in signal transmission via the spinothalamic pathway. This interpretation was based on finding an effect on LEP after laser stimulation of the lower limb, but not after laser stimulation of the upper limb. In the case of a generalized effect of tsDCS, it would have been expected to find an effect on LEP both after stimulation of the lower and the upper limb. Since they only found an effect on LEP after laser stimulation of the lower limb, they concluded that low-thoracic anodal tsDCS has a local effect on the spinal segments near the anode.

Thus, the effect on PREP could also be interpreted as a further indication for spinothalamic transmission, which could be attributed to PREP using CE having a nociception-specific property. This result is particularly important in light of the fact that PREP with CE are not considered nociceptor-specific, mainly due to their shorter latency compared to LEPs in the signal transmission via the spinothalamic pathway^12–14^.

However, it should be emphasized that we could not prove an inhibitory effect of anodal tsDCS on nociceptive stimuli, as was described previously and as we expected. In our study, N1P1-amplitudes of PREP after stimulation of the lower limb (area innervated by the sural nerve) remained almost unchanged (− 0.4 µV) after anodal tsDCS at the thoracic level, but decreased (− 8.5 µV) after sham stimulation (see Table 3). PREP after stimulation of the upper limb (area innervated by the medial nerve) also showed a decrease in N1P1-amplitudes, regardless of whether anodal or sham tsDCS was applied. Contrary to our findings, several previous studies on A-δ or C-fiber transmitted pain and/or evoked potentials found a decrease of pinprick pain^6^, pain evoked by cutaneous electrical stimuli^7,9^, or an increase in cold pain tolerance^2^ or pressure pain tolerance^10^ and especially on amplitudes of LEP^2,4^, although some studies also reported negative results in this regard^5,6^. This lack of decrease of the PREP amplitude after anodal tsDCS may rather indicate an opposite effect which could be interpreted as a suspended habituation or sensitization, respectively. Habituation of A-δ fibers was already reported using electrical stimulation with CE^21^ as well as when eliciting LEP^27^. But, since we have neither investigated habituation effects ourselves in this study, nor are there any published studies on habituation effects of PREP with CE, this hypothesis remains speculative.

The different effect of anodal tsDCS on PREP with CE could be explained on the one hand by the fact that CE directly activates A-δ and C-fiber in the superficial skin layers (short electric stimuli applied through concentric planar electrodes)^16^, while laser stimulation activates selectively A-δ and C-nociceptors ^14^. This may cause a difference in the nociceptive signal transduction. Nevertheless, it may be possible that A-ß-fibers are coactivated when using CE^12–14^, leading to different signal processing of A-δ or C-fiber input on the spinal level. Future studies comparing effects on PREP using low and high stimulation intensity (where Aß-activation is expected) may be informative.

Another possible explanation for our unexpected results could be that tsDCS affects neurons responding to different stimulation qualities differently, resulting in an increased transmission of nociceptive signals through Aδ-fibers and a decreased transmission of nociceptive signals through C-fibers. However, this cannot easily explain all of the previous study results, since LEP and pinprick pain both rely mostly on Aδ-fibers.

### Effect of low-thoracic anodal tsDCS on PREP-induced pain intensity

We observed a general increase of the subjective pain intensity caused by the cutaneous electrical stimuli using CE when eliciting PREP (evaluated by NRS) after anodal tsDCS compared to sham stimulation. However, this could not be shown separately for stimuli of the lower limb, so that we cannot recognize a connection to a local modulation of the low-thoracic tsDCS. Regarding the absolute values, the effect on PREP-induced pain intensity is very small, with an average difference between anodal and sham stimulation of fewer than 5 points on a NRS ranging from 0 to 100. While the results could be interpreted as a general sensitization to electrical stimuli by CE after anodal tsDCS, it should be noticed that this effect was generally weak and the absolute changes were very small. Descriptively, the effect seems to be somewhat stronger for the lower limb than for the upper limb (see Table 6), although this was not statistically significant in the ANOVA. This would be more consistent with the effect for the N1P1-amplitude, where there was no significant main effect of the stimulation type (sham vs anodal) on the N1P1-amplitude, but a significant effect of the interaction ‘limb’ x ’stimulation type’ on the N1P1-amplitude. Unfortunately, in the case of potentials elicited by nociceptive stimuli, the relationship between potential magnitude and induced pain intensity is not clear. While correlations have been demonstrated in previous work^16,28^, frequently observed dissociations between pain perception and potential magnitudes have been seen as indicating differences in the neural processes for the perception of pain stimuli and those for the brain activity elicited by the stimulus sampled by EEG^4^. Our results therefore remain difficult to interpret and can neither safely support the hypothesis of a local effect of tsDCS, nor be considered as evidence for a systemic effect.

**Table 6:** value after tsDCS minus value before tsDCS (for all dependent variables).

	foot		hand	
	sham	anodal	sham	anodal
N1P1 amplitude [µV]	-8.5 ± 10.5	-0.4 ± 15.7	-4.3 ± 14.5	-7.0 ± 12.1
N1 latency [ms]	1.9 ± 31.2	-2.5 ± 32.7	3.3 ± 19.9	2.9 ± 17.3
PREP-induced pain intensity [NRS (0–100)]	-2.6 ± 11.8	4.0 ± 9.7	-2.3 ± 10.0	0.76 ± 10.2
DT [mA]	0.06 ± 0.29	-0.02 ± 0.43	-0.01 ± 0.25	0.04 ± 0.19
PT [mA]	-0.01 ± 0.33	-0.01 ± 0.44	-0.07 ± 0.34	0.03 ± 0.2
NP amplitude SEP [µV]	0.1 ± 0.64	0.01 ± 0.62	0.06 ± 0.97	0.13 ± 0.62
N latency SEP [ms]	0.2 ± 3.89	-0.76 ± 3.49	0.44 ± 0.58	0.28 ± 1.1
Current intensity used for SEP [mA]	3.3 ± 4.5	1.7 ± 6.9	0.2 ± 2.3	-0.18 ± 2.1

### Limitations

Concerning the SEP, in our setup we only used one cortical recording electrode at a time and only evaluated the N20-P25-amplitude and N20-latency for the upper extremity and only the N30-P40-amplitude and N30-latency for the lower extremity. From this follows that we cannot rule out effects of tsDCS on other SEP values which could have been recorded using different montages, and we cannot rule out effects of tsDCS on SEP values associated with nociception. Furthermore, we cannot definitely ensure that in every subject the SEP stimulation electrodes were in the exact same position for every measurement. We tried to keep the variation low in this regard by checking the correct positioning of the electrodes before every measurement, but it is still likely that the positioning of the electrodes was not exactly the same during the course of the experiment. We found neither a carry-over effect nor a group effect on the current intensity of the SEP measurements, which indicates that this variation did not introduce systematic error into the SEP measurements. Still, it is possible that this variation reduced the power of our experiment regarding the SEP variables.

It should also be mentioned that the time interval between the end of tsDCS stimulation and PREP measurement for upper / lower limb varied depending on the group to which the subject was assigned (see Fig. 1). As an example: The time between end of tsDCS and start of PREP measurement of the lower limb varied between 0 min (group 2) and 30 min (group 3). We performed tests for effects of differences between these groups and found none, which makes a large difference depending on time of measurement less likely, but it is possible that weaker effects which might only have been measurable at a specific time after the stimulation were not detected.

The PREP parameters, especially the N1P1-amplitude, showed a considerable variability with standard deviations ranging from 36 to 51% of the mean for the N1P1-amplitude and from 13 to 23% of the mean for the N1-latency. These standard deviations lie within the range of previous studies on healthy subjects^15,22,29,30^, where standard deviations lay between 31 and 53% of the mean for the N1P1-amplitude and between 5 and 25% of the mean for the N1-latency.This underlines the high interindividual variability for PREP values, especially the N1P1-amplitude. On the other hand, a previous study found a high intraindividual test–retest-reliability with standard errors of measurement below 10% of the mean both for the N1-latency and for the N1P1-amplitude^31^.

An important limitation of this interpretation is the scarcity of data on habituation effects during PREP using CE since this was so far only reported in one study^21^ under the condition that stimuli with a constant current intensity were applied.

Regarding the change of N1P1-amplitude and NRS value during a session, there are some extreme values that fall outside of the range of 1.5× the interquartile distance, which is often used as a method to find possible outliers. Another method commonly used is the z-score-method. By this method, all of the possible outliers have a z-score of less than 3. We found no specific reason (e.g., technical errors) to exclude any of these data points. As can be seen from Figs. 2 and 3, median and mean values are generally very similar, which also makes it less likely that the results are driven by a few extreme values. Nevertheless, we cannot definitely exclude that the decision to exclude some or all of these extreme values, or some or all subjects with extreme values, may have changed the results of our study. To provide a more complete view of the results, we created versions of Figs. 2 and 3 in which data points outside of 1.5x the interquartile distance are marked by their subject ID, and in which data points measured from the same subject are additionally connected by lines. These figures are available as supplementary information (Supplementary information 1, Supplementary information 2).

In our study, we used sham stimulation as control experiment. Additionally, to further explore the underlying mechanism other control conditions could also be used, such as applying cathodal tsDCS, applying tsDCS over other regions of interest or placing the return electrode at other locations. The additional use of a cervical electrode could have been more conclusive as to whether the effect of low-thoracic tsDCS was actually a local one. So far, we can only draw this conclusion on the basis of previous results.

In summary, we could show an effect of low-thoracic tsDCS on N1P1-amplitudes of PREP using CE which was not inhibitory as expected, but rather facilitating. The neurophysiological mechanisms underlying these contrasting effects remain unclear and could not be investigated in this study. However, the fact that there was an effect, in contrast to SEP, suggests that PREP using CE are not the product of a Aß-fiber activation, but can rather exhibit a nociception specificity. Future studies should directly compare the effects on measurements utilizing different types of painful stimuli^3–5^ and confirm the local modulation of synaptic efficacy at the spinal level by applying tsDCS at different spinal levels.

### Supplementary Information

Supplementary Information 1.

Supplementary Information 2.

## Data Availability

The data collected or analyzed during this study are available from the corresponding author on reasonable request.
